# Dynamic operation of optical fibres beyond the single-mode regime facilitates the orientation of biological cells

**DOI:** 10.1038/ncomms6481

**Published:** 2014-11-20

**Authors:** Moritz Kreysing, Dino Ott, Michael J. Schmidberger, Oliver Otto, Mirjam Schürmann, Estela Martín-Badosa, Graeme Whyte, Jochen Guck

**Affiliations:** 1 Cavendish Laboratory, Department of Physics, University of Cambridge, Cambridge CB3 0HE, UK; 2 Max Planck Institute of Molecular Cell Biology and Genetics, Dresden 01307, Germany; 3 Niels Bohr Institute, University of Copenhagen, Copenhagen 2100, Denmark; 4 Max Planck Institute for the Science of Light, 91058 Erlangen, Germany; 5 Department of Physics, Friedrich-Alexander-Universität Erlangen-Nürnberg, 91052 Erlangen, Germany; 6 Biotechnology Center, Technische Universität Dresden, 01307 Dresden, Germany; 7 Department of Applied Physics and Optics, Universitat de Barcelona, 08028 Barcelona, Spain

## Abstract

The classical purpose of optical fibres is delivery of either optical power, as for welding, or temporal information, as for telecommunication. Maximum performance in both cases is provided by the use of single-mode optical fibres. However, transmitting spatial information, which necessitates higher-order modes, is difficult because their dispersion relation leads to dephasing and a deterioration of the intensity distribution with propagation distance. Here we consciously exploit the fundamental cause of the beam deterioration—the dispersion relation of the underlying vectorial electromagnetic modes—by their selective excitation using adaptive optics. This allows us to produce output beams of high modal purity, which are well defined in three dimensions. The output beam distribution is even robust against significant bending of the fibre. The utility of this approach is exemplified by the controlled rotational manipulation of live cells in a dual-beam fibre-optical trap integrated into a modular lab-on-chip system.

## Abstract (web summary)

Transmitting spatial information through optical fibres is difficult because scalar high-order modes deteriorate. Here, the authors counter deterioration using adaptive optics to excite vectorial modes, achieving high-quality beams robust against fibre bending and use those to rotate cells in a laser trap.

## Graphical abstract

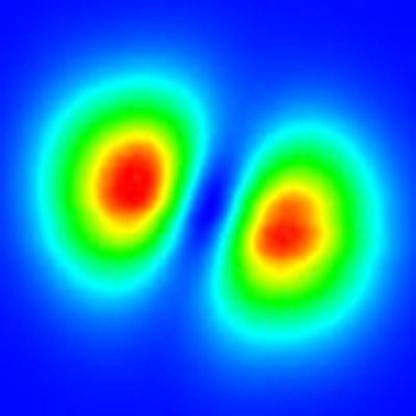

When an arbitrary light field enters an optical fibre, its propagation is most conveniently described on the basis of shape-invariant eigenfunctions of the propagation operator. The intensity distribution at any point along the fibre can then be obtained as the coherent sum of the initial modal fields multiplied by a complex phase factor. Because of non-trivial intermodal dispersion relations in optical fibres the dephasing between different modes leads to altered interference conditions giving rise to periodic changes of the intensity distributions even along an unperturbed fibre^1^ (Fig. 1a).

An often used, simplified description regards fibre modes as having a linear polarization, which can be variable in amplitude and sign, but not in orientation across the wavefront^2^. In-plane field amplitudes of these linearly polarized (LP) modes possess a scalar, orbital structure with radial and angular quantum numbers. This approximation is sufficient to explain the sudden occurrence of higher-order fibre modes with increasing fibre core diameter or refractive index step between core and cladding. Such an LP mode description has recently been used to measure and invert an empirical transmission operator between the two ends of a static multimode fibre^3^. LP modes, however, fail to predict the actual evolution of intensity profiles inside optical fibres. The reason for this rests in the violation of cylindrical fibre symmetry by these simplified wave functions (Fig. 1b left-hand side). Taking into account the symmetry of the problem requires a rigorous vectorial treatment of the wave equation in cylindrical coordinates. The solution, vectorial fibre modes (Fig. 1b right-hand side), are primarily classified by radial and angular quantum numbers as well. In addition, however, a spin quantum number is introduced, which describes the aligned or anti-aligned rotation of the polarization vector with the angular position on the wavefront. This photon spin gives rise to a splitting of propagation constants into a fine structure, which explains the decay of LP modes after half the beating length (for example, *l*_beat_≈36 cm for a Nufern 780HP fibre), which is on the order of 10^5^–10^6^ wavelengths^1^ (Fig. 1a, see also Methods). One ultimate result of this oscillatory mode dephasing is the practical difficulty in passing spatial information through optical fibres in a well-controlled way.

Recently, much progress has been made towards this goal by successfully applying adaptive optics methods, developed for aberration correction and scattering compensation^4^,^5^, to the shaping of the output beam of multimode fibres^3^,^6^,^7^,^8^,^9^,^10^,^11^,^12^ and photonic crystal fibres^13^. For this purpose, output responses to arbitrary sets of input fields (typically delta-peaks at the fibre entrance) have been probed experimentally, allowing for the construction of a transmission operator. This kind of careful empirical characterization of static multimode fibres allows both through-fibre imaging of microscale beads and their optical manipulation when located on a planar surface^7^,^10^. However, the lack of orthonormality between the many modes propagating in such 50-μm core fibres imposes practical limitations on achievable output fields, especially when trying to generate high-quality coherent fields that depend on the simultaneous excitation of adjacent fibre modes^7^. Although there is no fundamental reason why output beams of high modal quality should not be transmittable through multimode fibres, in practice this has been reported as very susceptible to noise, resulting in undesired interference effects in the generated fields.

Approaches, which define the output beams behind multimode fibres, need to suppress these unwanted interference effects between output modes either using iterative algorithms (mainly Gerchberg-Saxton type)^7^ or time-sharing, of which the latter finds a sensible application in the imaging through multimode optical fibres^11^. In all cases, however, the use of true multimode fibres requires the fibres to be kept static to avoid modal mixing, which limits the practical applicability.

Here we use a complementary approach for transmitting spatial information through optical fibres that results in well-defined, non-trivial beams propagating nearly diffraction-limited in three dimensions (3D). The approach, which in general has been demonstrated previously^1^,^3^, explicitly exploits the symmetry relation between the first few vectorial fibre modes, each of them being invariant under propagation. This allows for the selective excitation of pairs of vectorial modes that differ only regarding their spin quantum number and that are in phase periodically along the fibre (compare Fig. 1a). The beams emanating from the fibre end are near diffraction-limited and of LP mode type. Because their complex amplitude distribution closely resembles higher-order Laguerre–Gaussian beams, which are shape-invariant under free-space propagation^14^, the generated beams are highly suited for applications where the precise definition of light distribution in 3D is desired, such as in optical trapping. In addition, the use of a few-mode fibre renders the few selected modes largely robust against fibre bending, which adds an important practical dimension to this approach. We demonstrate its utility by using the beams generated for the controlled rotation of single biological cells in a fibre-based dual-beam laser trap incorporated into a lab-on-chip system. This could find far-reaching applications in the area of single-cell tomography, but also in general for the robust transmission of spatial information through optical fibres.

## Results

### Experimental realization

To excite individual modes experimentally (compare Fig. 2), either a 633-nm or a 1,072-nm single-mode laser beam, depending on the fibre used (see Methods), was expanded to a diameter of 8 mm to exploit the full resolution of the phase-only spatial light modulator (SLM; X10468-07, Hamamatsu, Japan), off which it was then reflected. A phase preserving telescope in 4*f*-configuration (best-form lenses L2 and L3, Thorlabs, UK) was used to project the spatially filtered and demagnified image of the field distribution into the back-focal plane of an aspheric coupling lens. Minor residual aberrations of the laser beam at this critical point were quantified with a Shack–Hartman wavefront sensor and pre-compensated with the SLM (see Methods and Supplementary Fig. 1). Precision coupling was likewise achieved by SLM scanning of the laser beam over the well-prepared fibre core (see Methods and Supplementary Fig. 2) in two orthogonal directions and by fitting the coupling ratio with an appropriate model function (see Methods and Supplementary Fig. 3). Lateral alignment precision better than 15 nm was maintained for >30 min (see Supplementary Fig. 3). Appropriate fibre lengths for particular pairs of vectorial fibre modes were chosen (see Methods for choice and stability).

### Mode rotation through few-mode fibre

**Figure 1:**
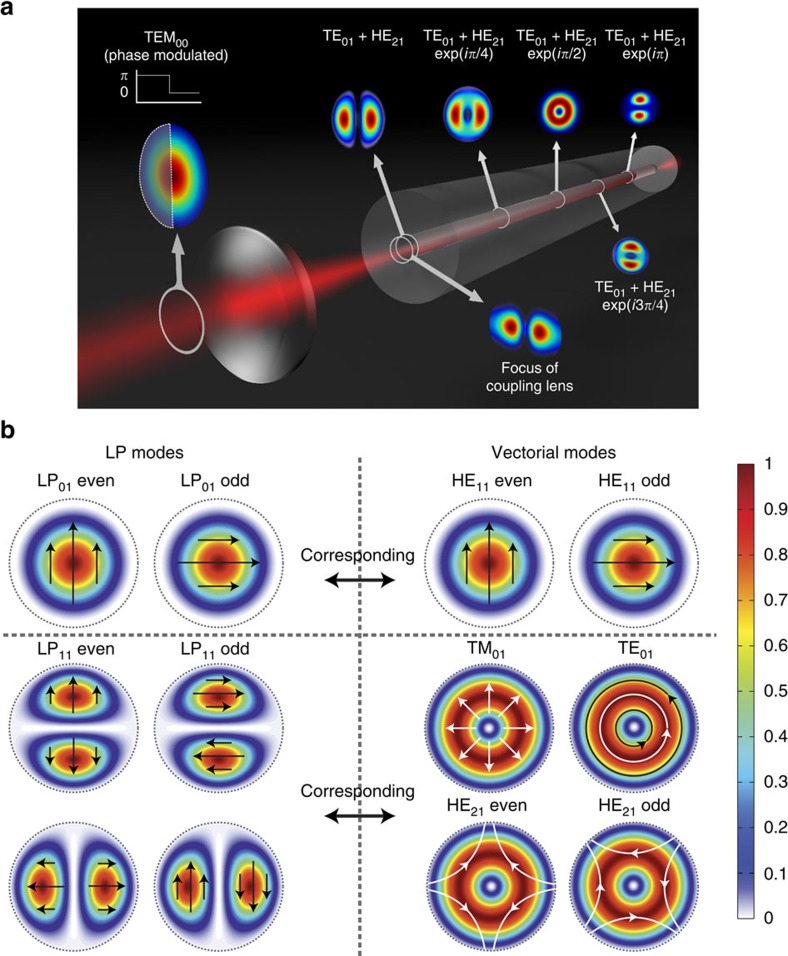
Illustration of the vectorial nature of fibre modes and its impact on mode dephasing. (**a**) A phase-modulated Gaussian intensity distribution (first from left) is imaged onto the fibre end and couples to a combination of the TM_01_ and HE_21_ vectorial fibre modes. This combination then experiences intermodal dephasing, which leads to a periodic image deterioration with propagation along the fibre. For example, the intermodal beating length for mixtures of the TM_01_ and HE_21_ modes upon propagation in a Nufern 780HP fibre is ~36 cm when operated at 633 nm. (**b**) Each of the scalar LP modes on the left side (the first two are shown) are linear superpositions of appropriate pairs of the vectorial fibre modes (right side). Small black and white arrows indicate polarizations. Colour scale indicates field intensities (in arbitrary units).

Imprinting symmetry-selective binary phase patterns on the SLM-diffracted wavefront prior to fibre coupling, previously suggested by von Hoyningen-Huene *et al.*^1^, yielded the specific emission of LP-like intensity beams from the far fibre end-face (Fig. 3). Because these beams rely on vectorial modes with orientational degeneracy caused by the symmetry of the fibre, field distributions at the fibre end-face can be readily rotated by the mere rotation of the input fields. Of note, a co-rotation of the polarization is required in the case of LP_11_ modes, since here an additional dephasing of transverse electric and transverse magnetic fields may occur (cf., Fig. 1b; Supplementary Fig. 4). Co-rotation ensures that the same linear combination of vectorial fibre modes is excited at any angle.

While the rotation of non-trivial intensity distributions behind fibres using pre-calculated holograms has been demonstrated before, the methodology presented here complements these techniques since the mode-specific excitation of fibre modes *a priori* determines the phase distribution of the output field. More importantly, the use of select eigenfunctions of the propagation operator in this few-mode fibre, rather than many as in previous reports using multimode fibres, renders this approach rather insensitive to even significant bending of the optical fibre (Fig. 4).

### Cell rotation in a dual-beam laser trap

Given the resulting 3D definition of the fibre output into free space, we demonstrate the beneficial application of this concept in the field of optical trapping of biological cells, objects much bigger than the wavelength of light. While single-beam gradient traps (commonly referred to as optical tweezers) are the established tool of choice to manipulate small biological samples down to the level of individual molecules via trapped colloids, the handle-free manipulation of entire cells with such tightly focused laser beams is difficult. A more appropriate approach to handle biological samples with sizes much in excess of the optical wavelength is offered by dual-beam laser traps^15^ most conveniently implemented using optical fibres^16^. Here, axial trapping stability is guaranteed by two divergent, counter-propagating beams even for large^17^ and multicellular samples^18^,^19^. One attractive aspect of fibre-based dual-beam laser traps is that the trap can be flexibly integrated into lab-on-chip setups for the convenient delivery of objects to be trapped^20^,^21^,^22^,^23^,^24^. At this point the insensitivity of the rotated output beams to fibre bending (Fig. 4) is particularly welcome. Also, a fibre-based dual-beam laser trap is completely decoupled from the imaging optics, which renders it perfect for the contactless orientation of cells for tomographic microscopy purposes^25^,^26^.

Towards this end, we demonstrate for the first time the trapping and precise rotation of cells perpendicular to the optical axis of a microscope in a dual-beam laser trap, eliminating the need for any mechanical rotation^18^ or alignment^27^,^28^ of optical fibres or apertures^29^. Figure 5a illustrates the working principle of a fibre-based dual-beam laser trap, into which we successfully integrated the setup presented above. The shaping and rotation of one of the laser beams via the SLM enables the dynamic control over the rotational degree of freedom of trapped cells about the laser-optical axis. The reorientation of cells in the trap is due to their tendency to maximize the overlap between regions of high refractive index and areas of high field intensity^29^.

Red blood cells, exhibiting strong shape anisotropy with minimal internal structure, and HL60 cells, spherical cells with typical internal structure^30^, were chosen as test objects based on their distinct morphological characteristics. For both cell types stable orientation and controlled rotation around the laser-optical axis could be achieved (Fig. 5b). This shows that both anisotropic shape and heterogeneous internal refractive index distribution are individually sufficient to determine the orientation of cells in the asymmetric trapping laser beam. The power in each of the beams can be <50 mW, so that the temperature in the trap is about 1.2 °C above ambient temperature and cell damage due to heating is avoided^31^. Of note, increasing the power leads to useful cell deformation (optical stretching)^32^, so that cell rotation and cell stretching can be combined in one setup.

## Discussion

A powerful future application is seen in combining this all-optical sample rotation technique with quantitative phase microscopy, thus enabling the determination of the 3D refractive index distributions of live cells^25^,^33^,^34^, or in widefield fluorescent imaging from multiple angles with subsequent image fusion, which would allow for significantly higher image quality and near-isotropic resolution^35^.

We conclude that adaptive optics can offer a convenient way to deliver non-trivial beams of high quality through optical fibres. It is evident that, due to the strongly reduced number of modes in the fibres employed here, intermodal coupling is much less of an issue than in the multimode fibres extensively studied before^3^. We further demonstrate the beneficial use of the advancements presented by the rotational alignment of live cells, a prerequisite for a multitude of single-cell tomographic microscopy techniques. The robustness of our approach against fibre bending permits the convenient integration of optical cell rotation with microfluidic lab-on-chip systems for convenient cell delivery. The combination of robust fibre optics with a small and modular microfluidic chip renders the system ready to be used with any microscope system. This aspect sets it apart from previous dual-beam laser trap micromanipulation approaches using free-space optics^17^,^36^ that do not easily permit this. More laboratory applications and completely novel approaches involving the delivery of spatial information through fibres, possibly extending the information content in fibre-based communication^37^,^38^, seem possible.

## Methods

### Aberration correction

Residual aberrations of the non-modulated beam in the back-focal plane of the coupling lens after alignment were conveniently corrected by displaying the appropriate Zernike polynomial on the SLM. The resulting root-mean-square flatness of the wavefront was better than 0.025 *λ* as measured by a Shack–Hartman wavefront sensor (Supplementary Fig. 2). Aberrations of the fibre-coupled light field may also result from a not perfectly planar fibre end-face. To avoid unwanted coupling between modes, the fibre ends were manually polished to a root-mean-square flatness of <5 nm (Supplementary Fig. 3) in some experiments.

### Fibre choice and preparation

The excitation of higher-order modes in single-mode fibres requires the use of light with appropriately shorter wavelength than the intended one. We used three kinds of commercial single-mode fibres (Nufern 780HP, 1060XP and 1310B-HP) and two different laser sources (5 mW HeNe laser, 05-LHP-151, Melles-Griot, UK for 633 nm or 5 W Ytterbium-doped fibre laser, YLG-1070-5LP, IPG Photonics, UK for 1,072 nm). Table 1 shows the different fibre and wavelength combinations and calculated *V*-parameters, which predict the guided mode groups (LP_01_ only for *V*<2.405, LP_01_ and LP_11_ for 2.405<*V*<3.83 and LP_01_, LP_11_ as well as LP_21_ for *V*>3.83). All predicted mode groups could be experimentally excited and rotated through the fibre as indicated in the two rows on the right-hand side. In particular, the excitation and rotation of the two pure LP-type modes shown in Fig. 3 was conducted using a Nufern 1310B-HP at 633 nm. The same fibre type operated at 1,072 nm was used for the optical trapping experiments, as the smaller number of guided modes ensured the mode profile to be more stable under rotations.

For stably holding and rotating optically trapped samples, a strong azimuthal asymmetry in the fibre’s output intensity profile is necessary. Thus, the phase relation between all vectorial fibre modes that are excited in the fibre has to be an integer multiple of 2*π* at the end of the fibre, as this will produce the ideal double-lobe LP_11_ mode structure, which has initially been coupled into the fibre, without any donut-like contributions (see Fig. 1a for the effect of intermodal dephasing on the fibre’s intensity profile). Hence, we developed a simple and reproducible iterative method that allows us to determine the fibre length *L*_fibre_ to fulfil this condition: while projecting the desired mode onto one end-face, the opposite end is shortened in steps of ~5 mm until the output intensity profile resembles the LP_11_ (that is, the desired, undistorted) mode shape when being inspected by eye or an infrared viewer. Then the coating is removed from the last 5–10 mm and the fibre is fixed to a metal plate. This allows the mode-mixture in the fibre output to be evaluated with a beam profiler while tiny pieces of the fibre’s end are chopped off with a razor blade until the wanted modal content is achieved.

### Fibre-coupling optimization

Fibre coupling into the few-mode fibre was optimized by scanning the laser beam over the fibre end-face in two orthogonal directions using the SLM. The measured coupling efficiency (Supplementary Fig. 4) was fitted with an appropriate function taking into account numerically evaluated coupling efficiencies^18^. The centre of these symmetric fits indicated the centre of the fibre core. The co-rotation of the polarization using a rotating half-wave plate (HWP) typically results in the orbiting of the laser focus on the fibre end-face around the optical axis due residual deviations from perfect surface parallelism. The radius of this orbit was determined to be 65 nm (Supplementary Fig. 4). The average deviation of the determined focus centres from this ideal orbit during rotation of the HWP indicates a 13-nm stability during the 30-min calibration procedure (Supplementary Fig. 4). The deviations introduced by the HWP rotation were compensated for by a fine-adjustable phase mirror implemented on the SLM.

### Optical trap

**Figure 2:**
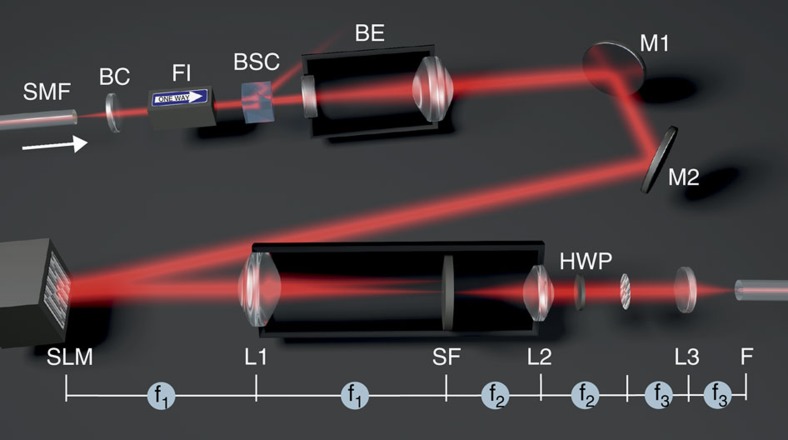
Schematic of the experimental setup. A laser beam (either HeNe laser, *λ*=633 nm, or Ytterbium-doped fibre laser, *λ*=1,072 nm) passes through a beam collimator (BC), Faraday isolator (FI) and beam-splitting cube (BSC), and is then expanded in a beam-expander (BE) to cover the entire active area of the spatial light modulator (SLM). A phase profile, appropriate to excite the desired modes, is then imprinted onto the beam at its reflection from the SLM, the beam diameter is reduced via a telescope of lenses L1 and L2 and finally coupled into the few-mode fibre by the lens L3. The zeroth diffraction order beam reflected off the SLM is blocked with a spatial filter (SF). For some modes it is necessary to rotate the polarization with a half-wave plate (HWP). For trapping experiments, the unmodified laser beam is split off with the BSC to form the second beam in the dual-beam trap (its coupling into a single-mode fibre (SMF) is not shown).

**Figure 3:**
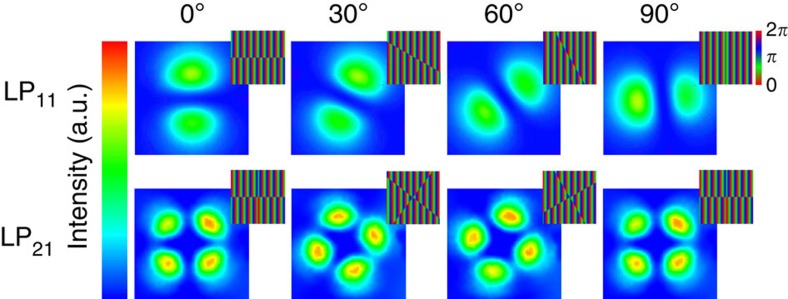
Rotational control of modes through an optical fibre with a spatial light modulator. The image sequences show successive mode orientations of the laser beam (*λ*=633 nm) emitted from the optical fibre (Nufern 1310B-HP) for an LP_11_ mode (top row) and an LP_21_ mode (bottom row). Callouts show the corresponding binary phase-modulating holograms displayed on the spatial light modulator. The four discrete orientations are exemplarily selected from a continuously varied angular orientation. See also Supplementary Movies 1 and 2.

**Figure 4:**
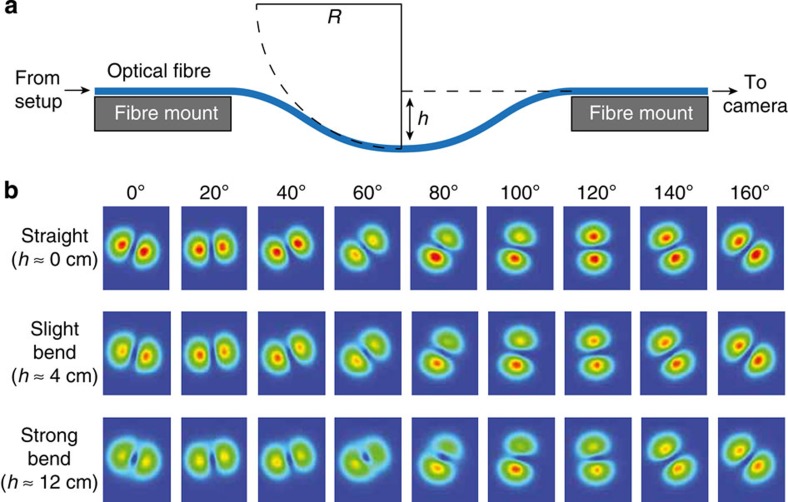
Insensitivity of mode rotation to fibre bending. (**a**) Schematic illustrating the experimental setup. An LP_11_ mode is coupled into a few-mode fibre and the output beam is monitored by a camera. The fibre is freely suspended over a 45-cm gap. Bending of the fibre can be induced by moving the fibre mounts closer together so that the fibre sags by different amounts, *h*, resulting in an approximate curvature of 1/*R*. (**b**) Images of the output beam profiles as the input is rotated through 160° in 20°-steps for three different amounts of fibre bending. Mode rotations remain largely unaffected even in the slightly and strongly bent fibre configurations (that is, sagging by 4 and 12 cm, respectively) and still provide sufficient trapping potential for cell rotation.

**Figure 5:**
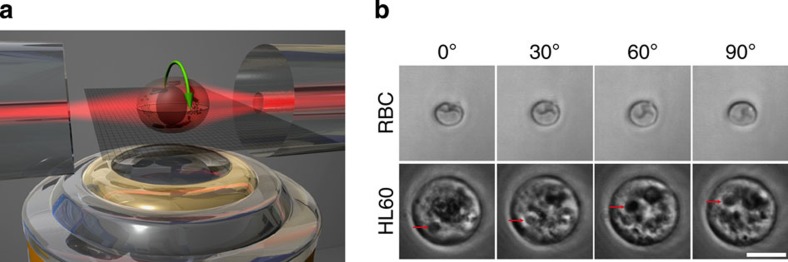
Rotation of biological cells in a fibre-based dual-beam laser trap operated beyond the single-mode regime. (**a**) Schematic illustrating the basic setup of the trap. Two opposing, coaxially aligned optical fibres emit a near-Gaussian beam (single-mode fibre, from left) and a rotational orientation-enforcing LP_11_ type beam (few-mode fibre, from right). The orientation of the LP_11_ type beam on the right side is controlled by the adaptive manipulation of wavefronts with the SLM prior to fibre coupling. (**b**) Image sequence showing the precise orientation of a red blood cell (top row) and a spherical HL60 cell (bottom row) between 0° and 90°. Scale bar, 10 μm. To avoid cell damage, the wavelength was *λ*=1,072 nm used with a Nufern 1310B-HP fibre. Arrows indicate object features, which help track the rotation (see also Supplementary Movies 3 and 4).

To form a dual-beam laser trap, the scattering force from the anisotropic laser beam discussed so far was balanced by a second, counter-propagating laser beam with a rotationally symmetric Gaussian beam profile. That second trapping laser beam was generated by splitting the laser beam (*λ*=1,072 nm) in the setup with a beam splitter (see Fig. 2) and then coupling it into a normal single-mode fibre (Nufern 1060 XP). The two optical fibres were mounted as described elsewhere^21^ (see also Supplementary Fig. 5). It should be noted that mode dephasing, even at perfect fibre length, can result from bending, twisting or compressing the fibre, which induces birefringence^39^, in principle. However, the setup described seems to be relatively robust against even considerable bending (*cf.* Fig. 4).

### Preparation of biological cells

Human red blood cells and cells from a human leukaemia cell line (HL60/S4) were used as typical biological cells. Red bood cells were prepared by diluting 1 μl of fresh blood from a healthy male donor in 10 ml isotonic phosphate-buffered saline solution containing 1 p.p.t. EDTA to avoid blood coagulation. HL60 cells (gift from Ada and Donald Olins, University of New England) were cultured according to a well-established protocol^40^. Briefly, the culture medium was RPMI 1640 containing 10% heat-inactivated fetal bovine serum, 0.3 g l^−1^ L-glutamine, and 1% penicillin/streptomycin at 37 °C and 5% CO_2_. Cells were passaged every 2–3 days and diluted to concentrations of ~10^5^ ml^−1^ for experimental use.

## Author contributions

M.K. and J.G. conceived the basic idea of an SLM-controlled optical cell rotator. M.K. and M.J.S. elaborated the theoretical aspects of the work. M.K. planned, supervised and supported the experimental implementation. D.O., M.J.S. and M.S. built the experimental setup. O.O., E.M.-B. and G.W. supported the SLM implementation and programming. M.J.S. performed the fibre-mode characterization and optimization experiments and D.O. performed the cell rotation experiments. M.K., D.O., M.J.S., G.W. and J.G. wrote the manuscript. The project was organized and coordinated by M.K. and J.G.

## Additional information

**How to cite this article:** Kreysing, M. *et al.* Dynamic operation of optical fibres beyond the single-mode regime facilitates the orientation of biological cells. *Nat. Commun.* 5:5481 doi: 10.1038/ncomms6481 (2014).

## Supplementary Material

Supplementary Information Supplementary Figures 1-5.

Supplementary Movie 1 Rotation of an LP11 mode through an optical fibre. The movie shows continuously changing LP11 mode orientations of a laser beam (λ = 633 nm) emitted from a few-mode optical fibre (Nufern 1310B-HP). The rotation is controlled by a spatial light modulator before the light is coupled into the fibre.

Supplementary Movie 2 Rotation of an LP21 mode through an optical fibre. The movie shows continuously changing LP21 mode orientations of a laser beam (λ = 633 nm) emitted from a few-mode optical fibre (Nufern 1310B-HP). The rotation is controlled by a spatial light modulator before the light is coupled into the fibre.

Supplementary Movie 3 Optical rotation of a red blood cell. A red blood cell is trapped in a fibre-based dual-beam laser trap (fibre ends to the left and right, outside of the field of view). The rotation of an LP11 mode emitted from one of the fibres causes the continuous re-orientation of the cell in the trap around an axis perpendicular to the imaging axis.

Supplementary Movie 4 Optical rotation of a white blood cell. A spherical white blood cell (HL60) is trapped in a fibre-based dual-beam laser trap (fibre ends to the left and right, outside of the field of view). The rotation of an LP11 mode emitted from one of the fibres causes the continuous re-orientation of the cell in the trap around an axis perpendicular to the imaging axis. The cell is rotated forwards and backwards to show flexibility.

## Figures and Tables

**Table 1:** Fibre types, *V*-parameters and observable mode groups.

	***V*** **633 nm**	***V*** **1,072 nm**	**Observable mode groups (633 nm)**	**Observable mode groups (1,072 nm)**
Nufern 780 HP	2.58	1.64	LP_01_, LP_11_	LP_01_
Nufern 1060 XP	3.50	2.06	LP_01_, LP_11_	LP_01_
Nufern 1310B-HP	4.79	2.82	LP_01_, LP_11_, LP_21_	LP_01_, LP_11_

For the three different fibre types and two different wavelengths (633 nm and 1,072 nm) used in the experiments, the first two columns show the *V*-parameters calculated and the second two columns the observed mode groups excited in the fibres.
