# Supplementary material for: Dynamic operation of optical fibres beyond the single-mode regime facilitates the orientation of biological cells
Source: Nat Commun. 2014 Nov 20;5:5481. doi: 10.1038/ncomms6481 (PMC4263128; doi:10.1038/ncomms6481)
Supplement: Supplementary Information — Supplementary Figures 1-5. [file ncomms6481-s1.pdf]

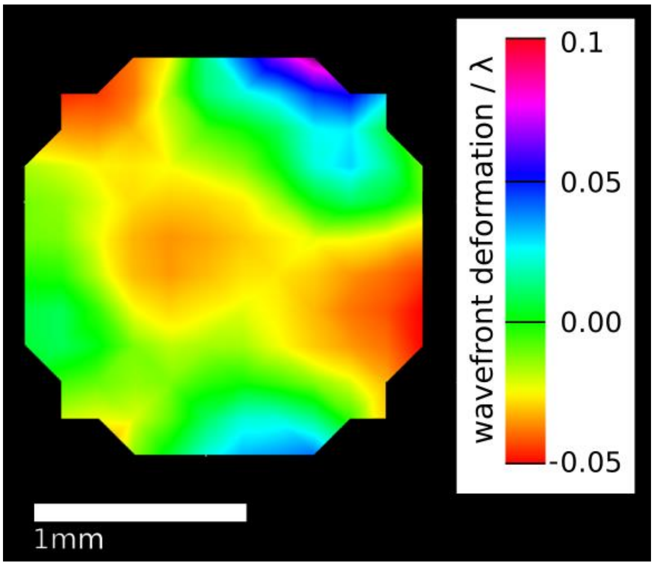

Supplementary figure 1 | Wavefront correction. Wavefront profile in the back focal plane of the coupling lens after alignment and minimization of residual aberrations, measured with a Shack-Hartman wavefront sensor. The route-mean-square deviation from a flat wavefront is below 0.025 λ.

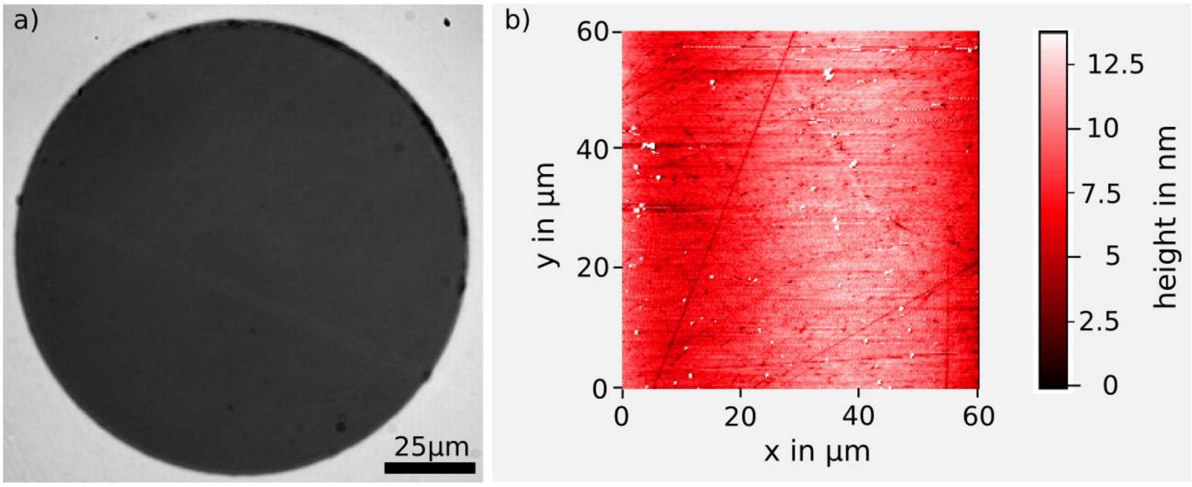

Supplementary figure 2 | Polished fibre end-face. Reflection light microscopy (a) and atomic force microscopy (b) images of typical polished fibre end-faces. The RMS roughness of the end face according to the AFM image was below 5 nm.

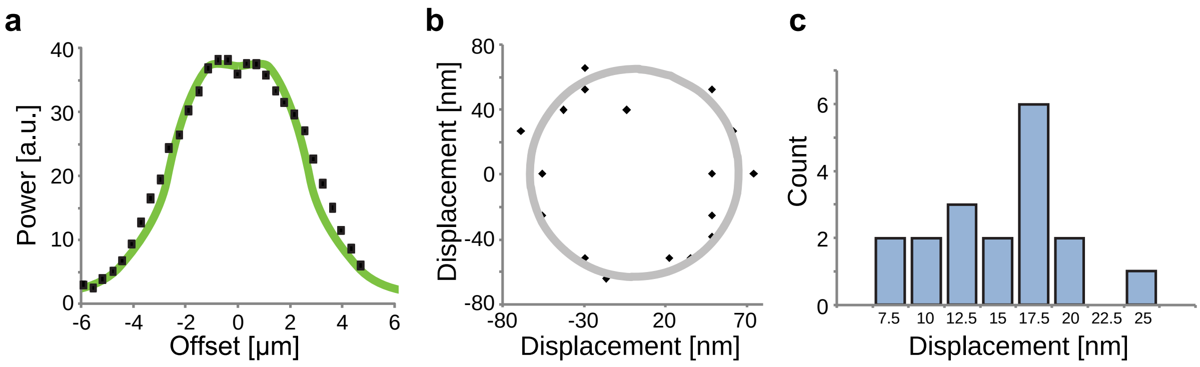

Supplementary figure 3 | Optimisation of fibre coupling. a) Laser power coupled into the few-mode fibre as a function of the lateral offset. b) Measurement of the deviation of the laser focus from the optical axis before fibre coupling due to the rotation of the polarization-adjusting half-wave plate. c) Radial distance of the laser foci from the ideal circular orbit resulting from the half-wave plate rotation shown in c). During the 30 min, which it took to obtain the measurement points shown in c), the real position of optimum coupling deviated slightly from the ideal orbit. The mean distance indicates an RMS centring stability of 13 nm over the 30 min.

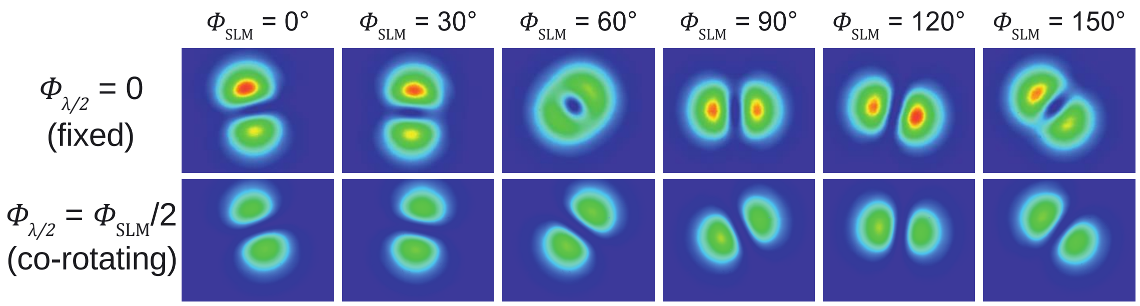

Supplementary figure 4 | Influence of the polarization co-rotation during SLM-induced rotation of the LP11 mode. Top row: Without co-rotation of the polarization using a half-wave plate a de-phasing of transverse electric and transverse magnetic fields is observed. Bottom row: With co-rotation of the half-wave plate the de-phasing is gone. Wavelength λ = 633 nm with Nufern 1060-XP fibre.

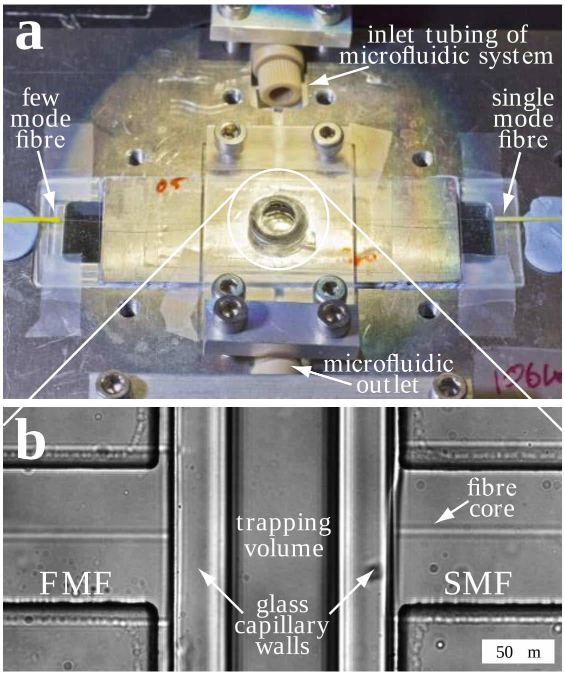

Supplementary figure 5 | Experimental trapping setup. a) Overview of the fibre alignment and the microfluidic capillary delivery system for the cell suspension on the microscope stage. b) Microscope image of the trapping region. The few-mode fibre (FMF) and the single-mode fibre (SMF) are coaxially aligned using an SU8-patterned surface (visible in the four corners). The fibre ends are moved onto the outer sides of a square glass micro-capillary, which runs perpendicularly from top to bottom.
